# 

**Published:** 1853-04

**Authors:** 


					﻿THE
WESTERN JOURNAL
OF
MEDICINE AND SURGERY.
EDITED KT
LUNSFORD P. YANDELL, M. I).,
raor««n,» at i>hv«iolo«y ano rATBOLoncAr. anatomy in this I'Nivratmv or i«chhi.i,».
ASO
THEODORE S. BELL, M. D.
CLX.—-APRIL, 1853.
THIRD SERIFS
Vol. XI...No. IV.
LOUISVILLE, KY:
PUBLISHED MONTHLY BY PRENTICE HENDERSON,
PROPRIETORS AND PRINTERS.
1 853.
				

